# Rapid *in situ* mutation detection in extracellular vesicle DNA

**DOI:** 10.20517/evcna.2024.69

**Published:** 2025-02-17

**Authors:** Md Mofizur Rahman, Lixue Wang, Yundi Chen, Md Motiar Rahman, M Oli Al Islam, Luke P. Lee, Yuan Wan

**Affiliations:** ^1^The Pq Laboratory of BiomeDx/Rx, Department of Biomedical Engineering, Binghamton University, Binghamton, NY 13902, USA.; ^2^Department of Radiotherapy, The Second Hospital of Nanjing, Nanjing University of Chinese Medicine, Nanjing 210003, Jiangsu, China.; ^3^Department of Chemistry, Binghamton University, Binghamton, NY 13902, USA.; ^4^ARCIM Institute, Filderstadt-Bonlanden 70794, Germany.; ^5^Harvard Medical School, Harvard University, Boston, MA 02115, USA.; ^6^Department of Medicine, Brigham and Women’s Hospital, Boston, MA 02115, USA.; ^7^Department of Bioengineering, University of California, Berkeley, CA 94720, USA.; ^8^Department of Electrical Engineering and Computer Science, University of California, Berkeley, CA 94720, USA.; ^9^Department of Biophysics, Institute of Quantum Biophysics, Sungkyunkwan University, Suwon 03063, South Korea.; ^10^Department of Chemistry and Nanoscience, Ewha Womans University, Seoul 03760, South Korea.; ^#^Authors contributed equally.

**Keywords:** Lung cancer, targeted therapy, DNA mutation, *EGFR*, CRISPR-Cas12

## Abstract

**Aim:** A PCR- and sequencing-free mutation detection assay facilitates cancer diagnosis and reduces over-reliance on specialized equipment. This benefit was highlighted during the pandemic when high demand for viral nucleic acid testing often sidelined mutation analysis. This shift led to substantial challenges for patients on targeted therapy in tracking mutations. Here, we report a 30-min DNA mutation detection technique using Cas12a-loaded liposomes in a microplate reader, a fundamental laboratory tool.

**Methods:** CRISPR-Cas12a complex and fluorescence-quenching (FQ) probes are introduced into tumor-derived extracellular vesicles (EV) through membrane fusion. When CRISPR-RNA hybridizes with the DNA target, activated Cas12a can *trans*-cleave FQ probes, resulting in fluorescence signals for the quantification of DNA mutation.

**Results:** This method enables the detection of *EGFR* L858R mutation in EV DNA within 30 min. Laborious extraction, purification, and other preparation steps for EV DNA are eliminated. The need for advanced data processing is also dispensed with. In a cohort study involving 10 healthy donors and 30 patients with advanced non-small cell lung cancer (NSCLC), the assay achieved a sensitivity of 86.7%, a specificity of 90%, and an accuracy of 87.5%.

**Conclusion:** The limit of detection of our Cas12 assay was ~ 8 × 10^5^ EVs, corresponding to a mutation allele frequency (MAF) of ~ 10%. The MAF in late-stage cancers varies widely but often falls within 5%-50%. Therefore, without amplification of targets, this Cas12 assay can detect mutations in patients with advanced lung cancer. Future advancements in multiplex and high-throughput mutation detection using this assay will streamline self-diagnosis and treatment monitoring at home.

## INTRODUCTION

Lung cancer is a major global health issue, both in terms of incidence and mortality. According to the World Health Organization (WHO): Lung cancer is the second most common cancer worldwide, accounting for approximately 11.6% of all new cancer cases. In 2020, there were an estimated 2.2 million new cases globally. Lung cancer remains the leading cause of cancer-related deaths worldwide, with approximately 1.8 million deaths in 2020. This makes it the most deadly cancer globally, responsible for around 18% of all cancer deaths. Incidence rates are higher in developed countries, particularly in regions with high smoking rates, such as the United States, Europe, and parts of Asia^[1,2]^. However, lung cancer rates are rising in many low- and middle-income countries, particularly among women and nonsmokers. Smoking is the primary risk factor that contributes to about 85% of cases. Both active smoking and secondhand smoke exposure are linked to lung cancer. Lung cancer is increasingly recognized in nonsmokers, with factors like genetics, exposure to environmental pollutants (e.g., radon, asbestos), and air pollution playing roles. Most cases occur in people aged 65 or older. Men historically had higher incidence rates, but rates among women have been rising, especially among nonsmokers. Non-small cell lung cancer (NSCLC) is the most common type, accounting for approximately 85% of lung cancers, and can be sub-categorized as Adenocarcinoma (more common), Squamous Cell Carcinoma, or Large Cell Carcinoma. Small cell lung cancer (SCLC) accounts for around 15% of lung cancers strongly associated with smoking, often diagnosed in late stage^[3,4]^. Various genetic mutations drive the development and progression of lung cancer. These mutations can be inherited or acquired over time, often as a result of smoking or environmental exposures. They are epidermal growth factor receptor (*EGFR*) Mutations, *KRAS* Mutations, anaplastic lymphoma kinase (*ALK*) Rearrangements, *BRAF* Mutations, *ROS1* Rearrangements, *TP53* Mutations, *PIK3CA* Mutations, and *MET* Amplifications. Early detection of lung cancer is critical for improving outcomes, but it remains a challenge due to the asymptomatic nature of early-stage disease. Several detection strategies are in use or under investigation, including Imaging Techniques [Chest X-ray, CT Scan (computed tomography), positron emission tomography (PET) Scan] Biomarker Testing [Liquid Biopsy (non-invasive), Tissue Biopsy] Sputum Cytology (mucus cough), Molecular Testing/personalized testing [PCR, next-generation sequencing (NGS), or fluorescence *in situ* hybridization (FISH)]^[5-8]^.

Growing evidence has shown the clinical relevance of extracellular vesicles (EV) in cancer. Recent studies have validated the use of EV analysis for (early) cancer diagnosis, treatment monitoring, and prognosis^[9,10]^. In the realm of EV-based cancer diagnostics, two primary technical approaches are currently used^[11]^. One approach involves on-chip analysis, where EVs are enriched on surfaces for the profiling of proteins in EV membranes. This method is convenient but merely analyzes membrane proteins. In contrast, the analysis of encapsulated EV cargo can be advantageous for EV-based cancer diagnosis^[12]^. However, the protective lipid bilayer of EVs impedes the access of detecting reagents to the cargo, rendering cargo detection challenging through the existing on-chip analysis. Alternatively, the second approach focuses on the isolation of EVs. Subsequently, multiple processing steps are required to properly extract and purify cargo molecules for molecular analysis. This approach enables comprehensive and accurate analysis. However, the labor-intensive sample preparation procedure significantly impedes analysis efficiency and promptness. Ideally, an on-chip detection approach that can circumvent cargo extraction and purification but interrogate wrapped cargo is highly desired. On the other hand, cancer patient services encountered significant challenges amidst the COVID-19 pandemic. Visits to cancer centers were minimized to reduce exposure risks, yet these protective measures also constrained cancer diagnosis and surveillance. This dilemma was apparent in patients undergoing mutation-targeted therapy, who could benefit from resistance tracking and timely medication adjustment^[13-15]^. In addition, genetic testing for cancers was crowded by the overwhelming COVID-19 nucleic acid tests, as both primarily rely on PCR and NGS^[16,17]^. In brief, the pandemic severely impacted cancer diagnosis and treatment monitoring^[14,18,19]^. This challenge highlights the critical importance of promptness and convenience in genetic testing.

The proposed assay offers multiple clinical applications, including early cancer detection, treatment monitoring, and as a companion diagnostic tool. For early detection, it identifies cancer-specific mutations, such as *EGFR* mutations, at an early stage through a non-invasive method using tumor-derived EVs, making it ideal for screening high-risk groups like smokers or those with a family history of cancer. This method allows for repeated screenings, increasing the chances of diagnosing cancers at localized stages. In monitoring treatment, the assay tracks mutation dynamics in EVs, providing insights into therapy effectiveness. A reduction in mutation signals indicates a positive treatment response, while persistent or new signals suggest resistance or relapse, eliminating the need for invasive biopsies. As a companion diagnostic, the assay aids in selecting targeted therapies by detecting actionable mutations, ensuring personalized treatment choices for patients. The implementation involves a streamlined workflow, including blood collection, EV isolation, and mutation analysis, without requiring complex equipment or specialized technicians. Validation through large-scale clinical trials and regulatory approval is necessary for its broader use. Future improvements may include adapting the assay for portable, point-of-care devices, enhancing its accessibility and integration into routine cancer care. Overall, the assay’s versatility and non-invasive nature make it a promising tool for cancer diagnosis, monitoring, and treatment selection.

**Figure 1:**
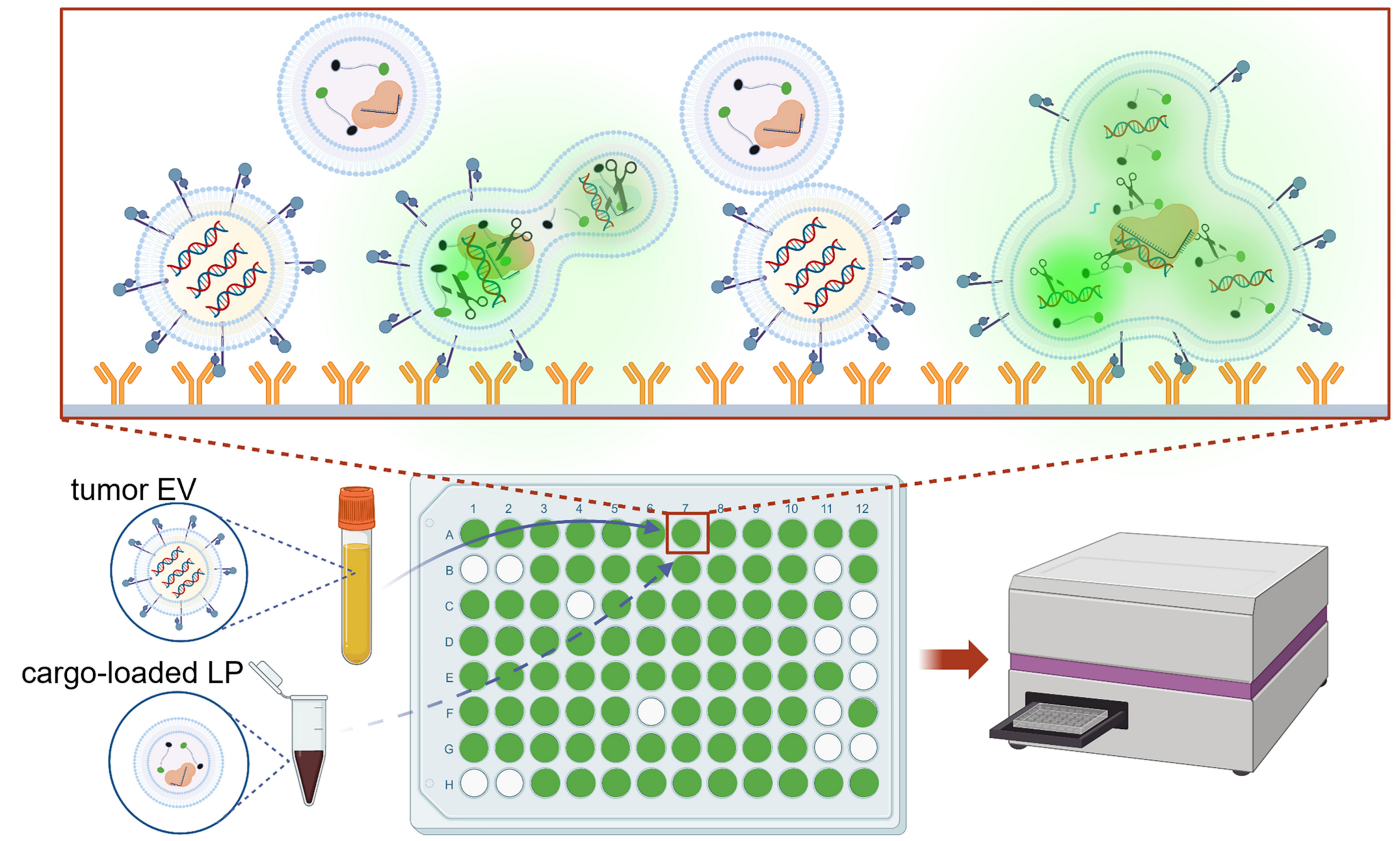
Schematic of CRISPR-Cas12a-based EV-DNA mutation detection. EVs in plasma are immunocaptured onto anti-EpCAM antibody-grafted surfaces, followed by membrane fusion with LPs containing Cas12a-crRNA and FQ probes. When crRNA hybridizes with the DNA target, activated Cas12a can cleave FQ probes, resulting in fluorescence signals. Therefore, DNA mutation can be qualitatively detected with a typical microplate reader. EV: Extracellular vesicle; FQ: fluorescence-quenching; LPs: liposomes.

Here, we report a PCR-, NGS-, and *in situ* amplification-free liquid biopsy using tumor-derived EVs for DNA mutation detection in 30 min^[15,20-22]^. Tumor-derived EVs in plasma are immunocaptured onto surfaces followed by membrane fusion with liposomes (LP) containing CRISPR-Cas12a and fluorescence-quenching (FQ) probes [Table 1]. The hybridization between DNA targets and complementary CRISPR RNAs (crRNA) triggers the cleavage activity of Cas12a, enabling the detection of DNA mutation through fluorescence signals emitted from shredded FQ probes [Figure 1].

**Table 1:** Sequences of oligomers used in this study

**Name**	**Sequence (5’ to 3’)**
crRNA for *EGFR* L858R	/AlTR1/rUrArArUrUrUrCrUrArCrUrArArGrUrGrUrArGrArUrGrCrCrCrGrCrCrCrArArArArUrCrUrGrUrGrArUrCrU/AlTR2/
FQ probe	FAM-TTTTAATTTT-IABkFQ

## METHODS

### Preparation of LP

LP were prepared using DOTAP,1,2-Dioleoyl-3-trimethylammonium propane, DMPC,1,2-dimyristoyl-sn-glycero-3-phosphocholine, DOPE, 1,2-dioleoyl-sn-glycero-3-phosphoethanolamine, and Cholesterol in molar ratios of 550:100:200:100, with 1 mol% NBD-PE, 1,2-dioleoyl-sn-glycero-3-phosphoethanolamine-N-(7-nitro-2-1,3-benzoxadiazol-4-yl) (ammonium salt), and 1 mol% Rhodamine-PE, 1,2-dioleoyl-sn-glycero-3-phosphoethanolamine-N-(lissamine rhodamine B sulfonyl) (ammonium salt). The thin film hydration method was employed to create a lipid layer, evaporating the organic solvent at 50 °C with a Rotavapor (30 rpm). Argon was used to further remove solvent in a fume hood for 1 h. The lipid film in the tube was vacuum-dried overnight. To make LP loaded with CRISPR-Cas12a complex and FQ probe, the lipid film was hydrated at room temperature, and the cargo was added to the tube. Control groups were prepared similarly. The mixture was briefly vortexed for 5 s, stirred at 100 rpm for 2 h at room temperature, and then left overnight at 4 °C. Finally, 100 nm LP were obtained by extruding the LP 20 times through a 0.1-micron polycarbonate membrane using an Avanti Mini-Extruder, and any free reagents were removed with a 300-KDa membrane filter following the manufacturer’s instructions.

### Cell culture and isolation of EVs

The human NSCLC cell lines NCI-H1975 (EGFR, T790M and L858R mutant) and NCI-H441(wild-type EGFR) were purchased from ATCC. Cells passed the mycoplasma contamination test, and were cultured in DMEM (Corning, USA). All media were supplemented with 5% (v/v) EV-depleted fetal bovine serum (FBS) (Thermal Fisher, USA), 100 units/mL penicillin, /mL streptomycin, and 1% non-essential amino acid. All cell lines were incubated at 37 °C with 5% CO_2_ and a 95% humidified atmosphere. Following seven passages, cells at 70% confluency were cultured in FBS-free medium for 48 h. The supernatant was then centrifuged at 2,500 g at 4 °C for 15 min, followed by a second centrifugation at 16,500 g for 20 min. Subsequently, the medium was filtered through a 0.22-μm pore filter, and the supernatant was ultracentrifuged at 100,000 g at 4 °C for 4 h.

### Nanosight measurement

LP and EV samples were suspended in 200 μL of PBS. Size distributions of LP and EVs were measured with Nanosight NS300 according to the manufacturer’s instructions. In brief, each sample was diluted 1,000-fold with PBS and manually injected into the chamber using a 1 mL syringe. The size distributions and concentrations of the EV and liposome samples were analyzed using a NanoSight NS300 instrument, with Nanoparticle Tracking Analysis Software (Malvern Instruments), and a capture time of 60 s for each sample.

### Zeta potential measurement

Zeta potential measurement was conducted using a Zetasizer Nano ZS system. Approximately 10 μL of the sample in 990 μL of deionized water (DI water) was transferred to a Malvern Clear Zeta Potential cell. Three independent aliquots were analyzed, and three measurements were taken for each aliquot.

### Transmission electron microscope

For transmission electron microscope (TEM), 5 μL of the sample was applied to a Formvar-coated copper grid (400-mesh) and incubated for 3 min at room temperature. Excess sample was removed with filter paper, and then the grid was negatively stained with 1% aqueous uranyl acetate for 1 min. After blotting dry, the samples were examined using a FEI Tecnai TEM at 100 kV.

### Förster resonance energy transfer assay

Förster resonance energy transfer (FRET) analysis was conducted using a TECAN Spark. For the NBD-chol/Rhod-PE pair, the excitation wavelength was set at 460 nm, and emission spectra were recorded from 500 to 700 nm. In a 96-well black-walled microplate, LP labeled with both NBD-PE and Rhod-PE were mixed with unlabeled EVs at liposome to EV ratios of 1:1, 1:3, and 1:5, totaling 50 µL. The EV-liposome fusion reaction mixture was incubated for up to 15 min, and fusion efficiency was determined by measuring the change in NBD fluorescence intensity before and after fusion. After fusion, 10% Triton X-100 (Sigma Aldrich, X100) was added to dissolve all vesicles and obtain the NBD fluorescence intensity *F*_∞_. The fusion efficiency was calculated using the formula: 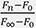 × 100%, where *F*_0_ and *F*_n_ represent the fluorescence intensities before and after fusion, respectively.

### *In vitro* Cas12a-based DNA mutation detection

The reaction mixture consisted of 50 nM Cas-12a, 50 nM crRNA, 10 U RNase inhibitor, and 40 nM FQ probe [Table 1]. This mixture was combined with the specified concentration of reaction buffer (50 µL containing 40 mM Tris-HCl at pH 7.5, 60 mM NaCl, and 6 mM MgCl_2_) and then incubated at 37 °C for 30 min. Fluorescent intensities were measured using a Tecan multi-plate reader.

### Fluorescence microscope

Isolated EVs were stained with PKH26 at 37 °C for 30 min. Excess dye was removed using an ultracel-30 membrane (Millipore, MRCF0R030). The labeled EVs were then collected and resuspended in PBS. Fluorescence images were captured using an Olympus IX83 microscope.

### Mutation detection in a microplate

In the Cas12a assay, a black-walled 96-well ELISA plate coated with anti-EpCAM (anti-epithelial cell adhesion molecule) antibodies was incubated at room temperature with 100 µL of purified EVs or plasma for EV capture. After three washes with PBS containing 0.05% Tween 20, sample wells were incubated with 50 µL of a reaction solution containing cargo-loaded LP. The fluorescent signal was then measured using a microplate reader. A positive EV [from NCI-H1975 (EGFR T790M and L858R mutant)] assay result was defined as a signal equal to or greater than a cut-off threshold, which was determined as the mean signal of the negative control samples [from NCI-H441(wild-type EGFR)] plus three times their standard deviation.

### Collection of plasma samples

Blood samples were obtained at the Second Hospital of Nanjing according to an institutional-review-board-approved protocol (IRB: 2016-LY-KT038). Samples were drawn into 10-mL EDTA (K2) tubes (Vacutainer; Becton Dickinson) from peripheral venipuncture. After centrifugation at 2,500 g for 15 min, plasma was collected and filtered using a 0.22-μm filter and stored at -80 °C until further analysis. The study included 10 healthy donors and 30 patients with late-stage NSCLC.

### Detection of DNA mutation by real-time quantitative PCR

Five µL of DNA was extracted from either the serially diluted samples containing mutated DNA (H1975 cell DNA) or crude human plasma. Each reaction was set up in a total volume of 50 µL, containing 1 × TaqMan™ Universal PCR Master Mix (without AmpErase™ UNG; ThermoFisher), 5 µL of DNA, and specific primers. PCR was performed using the Bio-Rad CFX96 Real-time system (Bio-Rad). The thermal cycling conditions were as follows: an initial incubation at 95 °C for 10 min, followed by 40 to 45 cycles of 95 °C for 10 s, 54.7 °C for 10 s, and 72 °C for 30 s. A final extension step was carried out at 72 °C for 10 min.

### Statistical analysis

Quantitative results were presented as mean ± SD. Student’s unpaired t-test was used to compare control samples (e.g., EVs from NCIH441) with experimental samples (e.g., EVs from NCI H1975). For multiple treatment groups, such as those with different doses of EVs (control, experimental, CRISPR-loaded LP), statistical significance was examined using one-way ANOVA. Different statistical software packages were also used in this study.

## RESULTS

### Strategies for designing and mechanistic features of the Cas12a-crRNA complex

**Figure 2:**
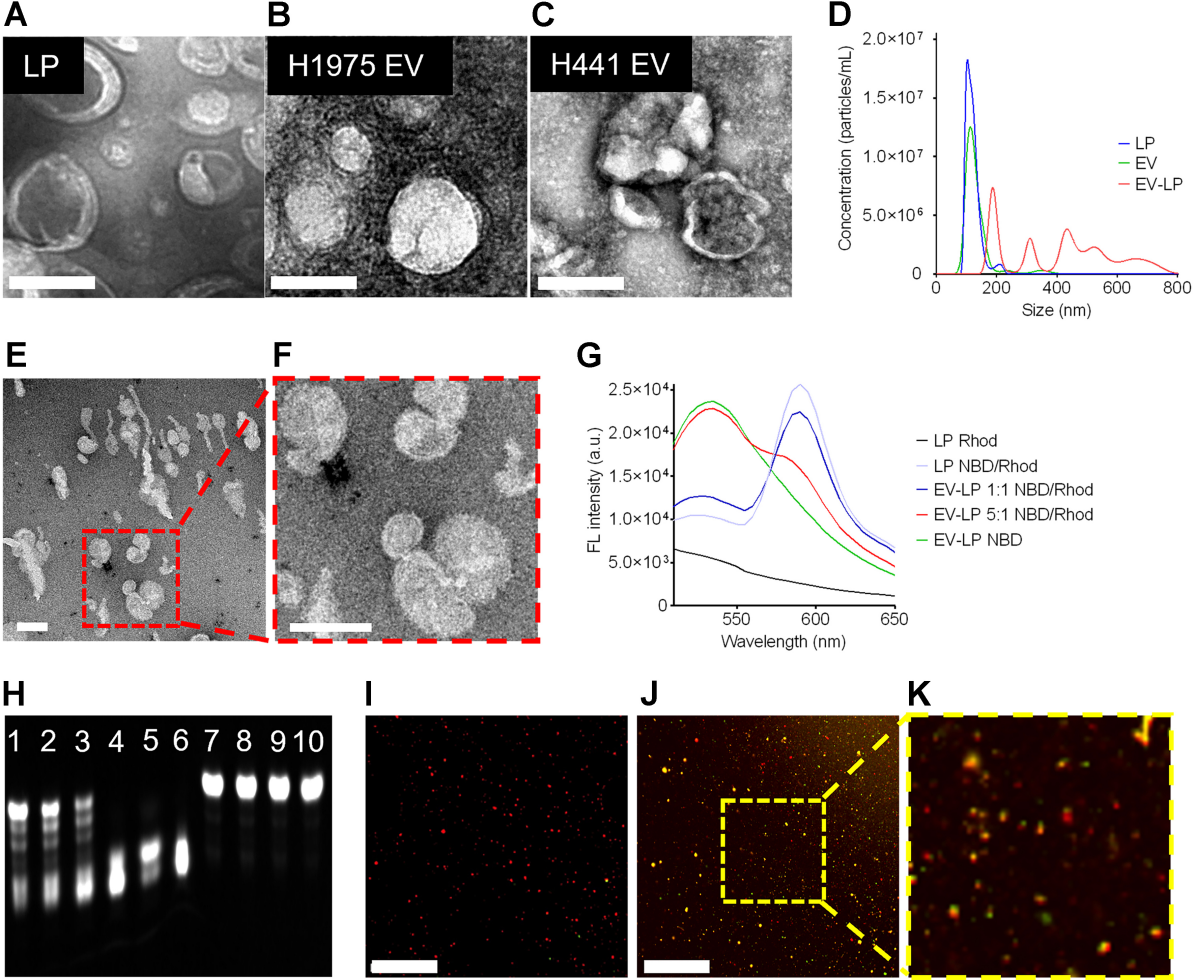
Characterization of EV-LP fusion and validation of ribonucleoprotein in mutation detection. (A-C) Electron microscope image of LP, H1975 EVs, and H441 EVs, respectively (scale bar: 100 nm); (D) Size distributions of LPs, EVs, and fused EV-LP; (E and F) Electron microscope image of EV-LP fusion; (G) Fluorescence signals of dyes-labeled LPs in the FRET assay; (H) Gel analysis of the FQ probes after cleavage (Lane 1-5: FQ probes were *trans*-cleaved by Cas12a in the presence of the L858R mutation; H1975 EV amount was 1 × 10^5^, 1 × 10^6^, 1 × 10^7^, 1 × 10^8^, and 1 × 10^9^, respectively. Lane 6: FQ probes were cleaved by Cas12a, which was used as a positive control. Lane 7: FQ probes were not cleaved by Cas12a in the absence of L858R mutation; H441 EV amount was 1 × 10^10^. Lane 8: FQ probes were not cleaved by Cas12a in the absence of EV DNA. Lane 9: FQ probes were not cleaved in the absence of Cas12a. Lane 10: FQ probes only; (I-K) Fluorescence co-localization analysis of fused EV-LP. (I) In the absence of the L858R mutation, only fluorescence signals emitted from PKH26 (red) were observed in H441 EV-LP (left; scale bar: 50 µm); (J) In the presence of the L858R mutation, dual fluorescence signals emitted from PKH26 and shredded FQ probes (green) were observed in H1975 EV-LP (right); (K) The insert shows a local zoom-in view. EV: Extracellular vesicle; FRET: förster resonant energy transfer; FQ: fluorescence-quenching; LP: liposomes.

Fusogenic LPs were specifically designed to deliver a Cas12a-crRNA complex, which is a key component of CRISPR-based gene editing, as well as FQ probes. The formulation of these LPs resulted in particles with an average size of approximately 100 nm. This size is particularly important because it is within the range of small EVs, which typically range from 30 to 150 nm. This size similarity helps facilitate the interaction between the LPs and the EVs, as they can more readily undergo membrane fusion, a critical aspect of the delivery process. The alignment of the LPs’ size with that of small EVs is illustrated in Figure 2A-D. The surface charge of LPs and EVs was quantified through zeta potential measurements. The zeta potential is a key indicator of the electrostatic properties of particles in solution. The average zeta potential of LPs, Cas12a-crRNA complex-loaded LPs, H1975 EVs, and H441 EVs were 71.02, 40.73, -46.31, and -44.25 mV, respectively. These LPs engaged in membrane fusion with EVs through electrostatic interactions, facilitating the delivery of their contents into the EVs and resulting in fused vesicles with sizes ranging from 200 to 800 nm. The complete fusion and fusion intermediate were visualized by electron microscopy images [Figure 2D-F]. After fusion, the average zeta potential of H1975 EV-LP and H441 EV-LP was -10.65 and -11.33 mV, respectively.

FRET assay also verified their fusion^[23,24]^. Modulating the EV-to-LP ratio, either by increasing or decreasing it, reduced FRET efficiency [Figure 2G], suggesting that membrane fusion scattered FRET dyes on liposomal membranes. FRET is a sensitive technique that detects interactions between fluorophores-molecules that emit light when excited. FRET occurs when two fluorophores labeled LPs, a donor (NBD-PE) and an acceptor (Rhod-PE), are in close proximity (typically within 10 nm). In the context of this experiment, FRET was used to measure the proximity of the liposomal membranes before and after fusion. The FRET results demonstrated a decrease in FRET efficiency when the ratio of EVs to LPs was altered. Specifically, either an increase or a decrease in the amount of LPs led to lower FRET efficiency and vice versa. This is because, as the LPs and EVs fused, the fluorophores initially on the liposomal membranes spread out across the merged vesicle membranes, reducing their ability to interact and transfer energy. This decrease in FRET efficiency suggests that the liposomal membranes were successfully fused, as the dye molecules were no longer in the same proximity on the separate membranes.

Nevertheless, the average fusion efficiency was determined to fall within the range of 50% to 90%. The assay was optimized with tumor EV model samples. Mutation detection specificity was confirmed before performing *in situ* DNA mutation detection for lung cancer. Gel electrophoresis verified concentration-dependent *trans*-cleavage of FQ probes into short fragments upon exposure to activated Cas12a in the presence of the L858R mutation. In contrast, this cleavage was absent in scenarios devoid of this mutation [Figure 2H]. In parallel, we determined that the average loading efficiency of Cas12a-crRNA and FQ probe was 89.22% and 94.67%, respectively. Next, fluorescence co-localization analysis confirmed the successful delivery of cargo by LPs to PKH26, Red Fluorescent dye-labeled EVs. Strong green fluorescence signals, generated from cleaved FQ probes, were detected in approximately 85% of H1975 EVs carrying the L858R mutation, which activates the CRISPR-Cas12a reaction for signal detection. In contrast, less than 0.1% of H441 EVs lacking the L858R mutation exhibited negligible green fluorescence signals [Figure 2I-K].

### Quantitative analysis of EV-derived DNAs for the detection of mutations in lung cancer

**Figure 3:**
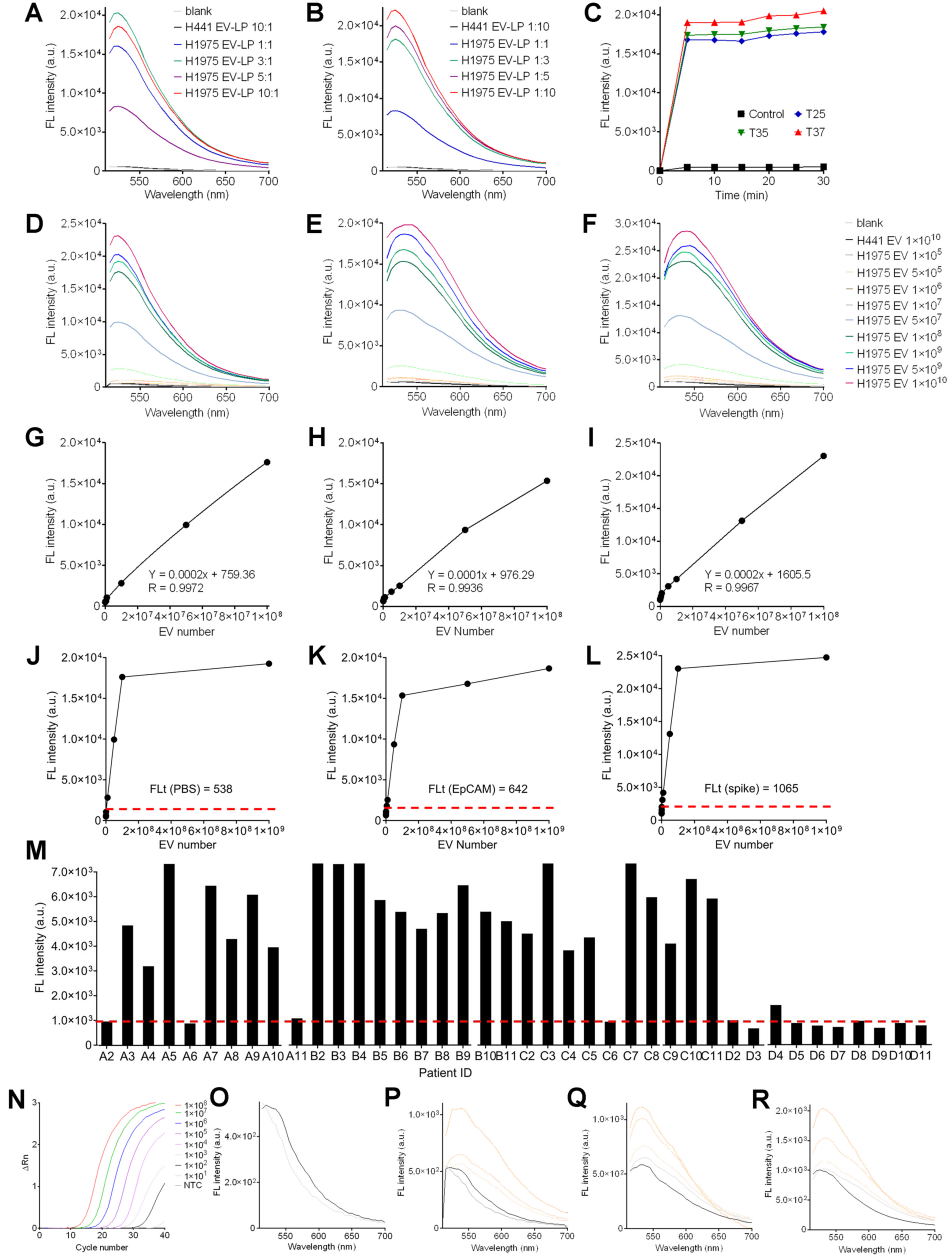
Characterization and optimization of Cas12a-based assay. (A and B) Quantitative fluorescence intensity detected from cleaved FQ probes in various EV-to-LP ratios. The reaction volume was 50 µL; (C) Quantitative fluorescence intensity detected from EV-to-LP ratio of 5:1 after 30-min incubation at 25, 35, and 37 °C, respectively. The reaction volume was 50 µL; (D, G, J) LOD of L858R mutation in H1975 EVs suspended in PBS; (E, H, K) LOD of L858R mutation in H1975 EVs suspended in PBS followed by immunocapture onto surfaces; (F, I, L) LOD of L858R mutation in H1975 EVs suspended in human plasma followed by immunocapture onto surfaces; (M) L858R mutation detection in patient’s plasma EVs. Thirty patients with stage-IV NSCLC and ten healthy volunteers were enrolled. The red dot line indicates the FLt of mutation detection; (N) Detection of L858R by using RT-qPCR. DNA fragments were extracted from various amounts of H1975 EVs, followed by RT-qPCR detection; (O, P, Q, R) showed a zoomed-in view of the blanks for (A) and (B). The zoomed-in view of low FL for (D-F) was presented in (P-R). FQ: Fluorescence-quenching; EV: extracellular vesicle; LOD: limit of detection; NSCLC: non-small cell lung cancer; RT-qPCR: real-time quantitative PCR; FLt: fluorescence threshold; FL: fluorescence.

We designed the CRISPR/Cas12a assay by incorporating specific target sequences to detect DNA mutations in EVs associated with lung cancer, along with dye-labeled single-strand DNA into a LP. To accomplish this, antibody-captured EVs were incubated with the liposome-loaded CRISPR, and the fluorescence signal generated by the fusion of LP and EVs was measured. EVs were isolated from both cell culture media and human plasma samples, and then captured using anti-EpCAM antibodies. *In situ* detections were performed using purified EVs from human NSCLC samples, specifically NCI-H1975 cells with the EGFR L858R mutation, and NCI-H441 cells with the EGFR wild-type as controls. These samples were subsequently analyzed through parallel *in situ* detection and RT-PCR assays [Figure 3]. The quantitative analysis using a microplate reader revealed that the signal intensity exhibited a proportional increase with the rising level of L858R copies, eventually reaching saturation [Figure 3A]. In this scenario, all Cas12a-crRNA complexes effectively engaged and bound to the targeted DNA. The Cas12a *trans*-cleaved a limited number of FQ probes. A parallel trend was observed when maintaining the L858R copies but increasing the quantity of Cas12a-crRNA complexes [Figure 3B]. We postulated that all mutant targets underwent *cis*-cleavage, while an excess of FQ probes in fused vesicles simultaneously experienced *trans*-cleavage. The findings indicated that the overall quantity of FQ probes in fused vesicles determined the signal intensity. Moreover, we observed that the reaction kinetics of Cas12a remained relatively consistent at 25 °C compared to those at 35 and 37 °C. Maximum signal intensities were detected after a mere 5-min incubation [Figure 3C]. To facilitate this study, we adopted 25 °C in the subsequent experiments. Notably, if conditions permit, 37 °C is indeed the optimal choice for mutation detection. The fluorescence threshold (FLt) and limit of detection (LOD) of this assay were measured. Each H1975 EV contains ~ 10.5 to 24.6 copies of DNA fragments harboring the L858R mutation. Various amounts of H1975 EVs were resuspended in PBS and subjected to detection using 2.5 × 10^8^ LPs [Figure 3D, G, and J]. The FLt was established at 538 arbitrary units (a.u.), and the LOD^PBS^ in solution was calculated to be 3.14 × 10^5^ H1975 EVs. Using the same experiment setup, we found that FLt and LOD^PBS^ on the EpCAM-coated plate surface were 642 a.u. and 6.27 × 10^5^ H1975 EVs [Figure 3E, H, and K]. In addition, we tested 5 µL of EV-spiked human plasma samples to simulate clinical conditions. The average immunocapture efficiency was 92.45% in 15 min. The FLt and LOD^Plasma^ on the anti-EpCAM antibody-coated plate surface was 1,065 a.u. and 8.3 × 10^5^ H1975 EVs [Figure 3F, I, and L]. Lastly, we assessed this assay using plasma samples collected from 10 healthy donors and 30 patients with late-stage NSCLC [Figure 3M and Table 2]. Cancer patients’ L858R mutation in circulating tumor DNA was verified with real-time quantitative PCR (RT-qPCR). We determined that the sensitivity, specificity, and accuracy of our assay were 86.7%, 90%, and 87.5%, respectively. Notably, mutation detection was fulfilled in 30 min. In comparison, RT-qPCR spent approximately two hours detecting mutation from ~ 8 × 10^5^ H1975 EVs [Figure 3N]. These findings demonstrate the feasibility, accuracy, and reliability of this assay.

**Table 2:** Clinical information of patients with stage-IV NSCLC

**Well**	**RT-qPCR**	**Cas12a-based detection**	**Sex**	**Age**
A2	L858R+	FN	M	68
A3	L858R+	TP	M	77
A4	L858R+	TP	M	55
A5	L858R+	TP	F	62
A6	L858R+	FN	F	58
A7	L858R+	TP	M	57
A8	L858R+	TP	F	52
A9	L858R+	TP	F	73
A10	L858R+	TP	F	64
A11	L858R+	FN	M	45
B2	L858R+	TP	M	28
B3	L858R+	TP	F	45
B4	L858R+	TP	M	75
B5	L858R+	TP	F	77
B6	L858R+	TP	M	51
B7	L858R+	TP	M	56
B8	L858R+	TP	M	58
B9	L858R+	TP	M	72
B10	L858R+	TP	F	70
B11	L858R+	TP	F	81
C2	L858R+	TP	F	70
C3	L858R+	TP	M	70
C4	L858R+	TP	M	65
C5	L858R+	TP	F	67
C6	L858R+	FN	M	73
C7	L858R+	TP	F	68
C8	L858R+	TP	F	56
C9	L858R+	TP	F	69
C10	L858R+	TP	M	48
C11	L858R+	TP	F	61
D2	L858R-	TN	M	54
D3	L858R-	TN	M	52
D4	L858R-	FP	M	63
D5	L858R-	TN	F	61
D6	L858R-	TN	M	49
D7	L858R-	TN	F	74
D8	L858R-	TN	F	56
D9	L858R-	TN	M	50
D10	L858R-	TN	M	33
D11	L858R-	TN	M	51

TP: True positive; FP: false positive; TN: true negative; FN: false negative; NSCLC: non-small cell lung cancer; RT-qPCR: real-time quantitative PCR.

### Model performance

**Figure 4:**
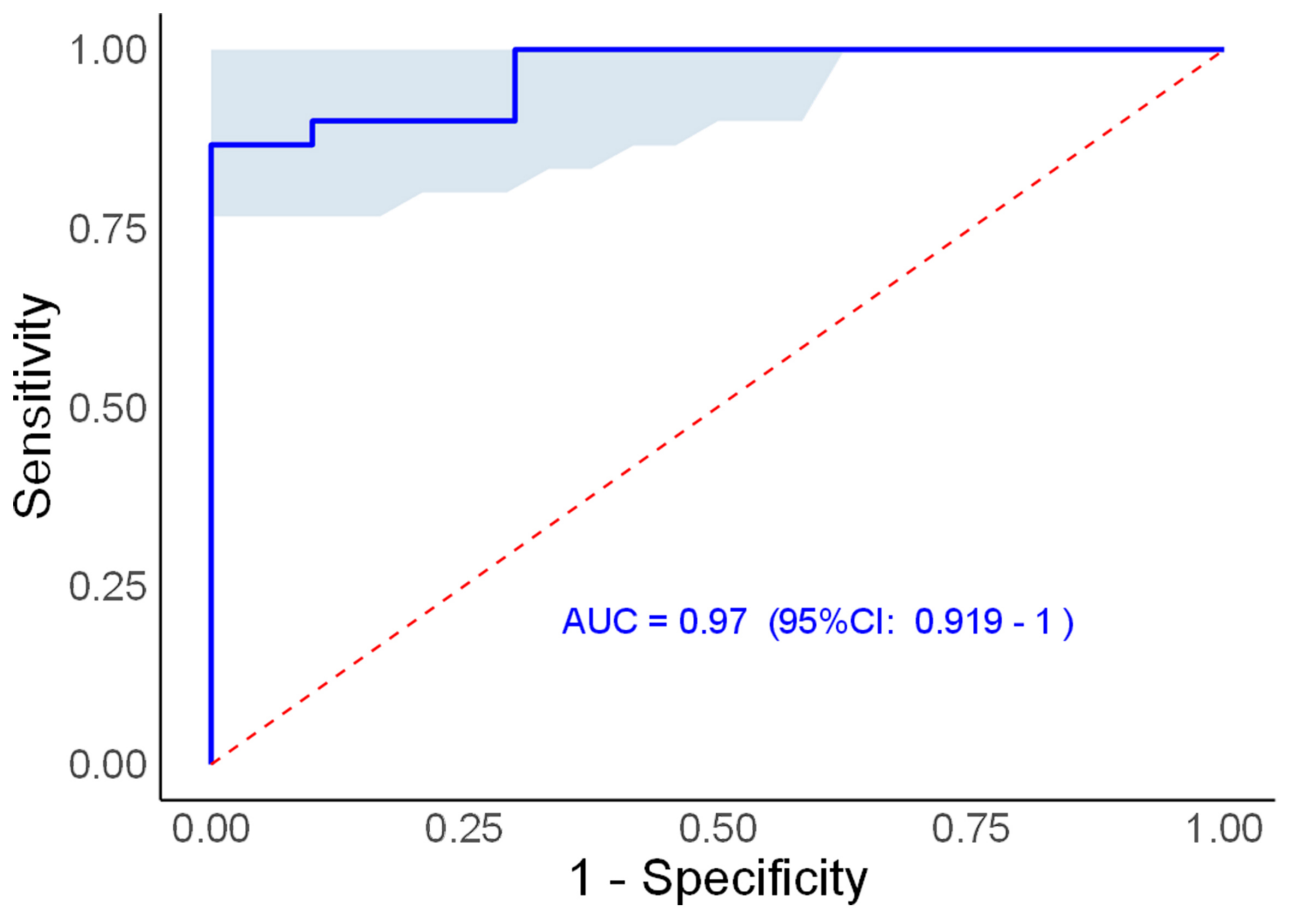
Performance of *in situ* cancer DNA mutation detection. The AUC, CI, and cut-off values are 0.97, 0.919-1, and 1,000, respectively, in the ROC curve analysis for the cancer mutation detection in EV. AUC: Area under the curve; ROC: receiver-operating characteristic; CI: confidence interval; EV: extracellular vesicle. *P* < .05. Performance of *in situ* cancer DNA mutation detection. The AUC, CI, and cut-off values are 0.97, 0.919-1, and 1,000, respectively, in the ROC curve analysis for the cancer mutation detection in EV. AUC: Area under the curve; ROC: receiver-operating characteristic; CI: confidence interval; EV: extracellular vesicle. *P* < .05.

The AUC measures the ability of a model to discriminate between classes - in this case, distinguishing between positive and negative instances (e.g., presence or absence of a disease). The AUC value ranges from 0 to 1, where: 0.5 indicates no discriminative power (random guessing); 1.0 represents perfect discrimination (the model correctly classifies every instance). Our proposed assay displayed an AUC of 0.97, indicating that the model is highly effective [Figure 4], with a 97% chance of correctly distinguishing between a positive and a negative case of mutation detection. This is considered an excellent performance in our proposed assay.

The confidence interval provides a range of values within which the true AUC is likely to fall, with a certain level of confidence (usually 95%). In this case, the confidence interval is between 0.919 and 1, meaning that we can be reasonably confident (95% confidence) that the actual AUC value lies within this range. The lower bound (0.919) still indicates strong model performance. The upper bound (1) suggests that, in the best-case scenario, the model could achieve perfect performance for detecting DNA mutation in cancer.

### Gender or age-specific bias of occurrence of these mutations

Gender-Specific Bias: The chi-square test (X^2^ = 2.7817, *P* = 0.4265) and Fisher’s exact test (*P* = 0.4764) indicate no significant association between gender (Sex) and the occurrence of the mutations. Both tests show *P*-values much higher than 0.05, leading to the conclusion that there is no evidence to support a gender-specific bias in mutation occurrence [Table 3].

**Table 3:** Gender- or age-specific biases of occurrence of detected mutations

	**Result**	**TP**	**FN**	**TN**	**FP**	**c (TP, FN, TN, FP)**
Sex	Total	26/26	4/4	9/9	1/1	40/40
	Male	12 (46.15%)	3 (75.00%)	6 (66.67%)	1 (100.00%)	22 (55.00%)
	Female	14 (53.85%)	1 (25.00%)	3 (33.33%)	-	18 (45.00%)
	Missing (%)	-	-	-	-	-
AgeX	Total	26/26	4/4	9/9	1/1	40/40
	Higher or equal to Med	16 (61.54%)	2 (50.00%)	2 (22.22%)	1 (100.00%)	21 (52.50%)
	Less than Med	10 (38.46%)	2 (50.00%)	7 (77.78%)	-	19 (47.50%)
	Missing (%)	-	-	-	-	-

TP: True positive; FP: false positive; TN: true negative; FN: false negative.

Age-Specific Bias: The age variable was originally continuous but was converted into a categorical variable using a median split: values higher or equal to the median were placed in one category, and values lower than the median in another. The chi-square test (X^2^ = 5.0751, *P* = 0.1664) and Fisher’s exact test (*P* = 0.1535) suggest no significant association between age (AgeX) and mutation occurrence. Similarly, the *P*-values are higher than 0.05, meaning there is no evidence of an age-specific bias in the mutation data [Table 3].

In short, the analysis demonstrates no evidence of either gender-specific or age-specific biases in the occurrence of these mutations in the provided dataset. The results suggest that mutation occurrence is not significantly associated with either variable, even after converting age into a categorical variable.

## DISCUSSION

RT-qPCR and NGS are standard diagnostic tools in clinical laboratories^[25-27]^. Both require specialized equipment and skilled staff^[28]^. To monitor the mutation status of patients undergoing EGFR-targeted therapy, RT-qPCR takes a minimum of two hours, and NGS takes at least two days [Table 4]. In contrast, this CRISPR-Cas12 assay does not demand specialized sample handling or complex data analysis^[29]^. It achieves mutation detection using a common microplate reader in 30 minutes, reducing the duration and exposure risks of vulnerable patients with advanced cancers in hospitals. The detection sensitivity and specificity are on par with those of RT-qPCR. Notably, this assay eliminates the need for *in situ* amplification of nucleic acids^[30]^, streamlining the entire detection process for increased simplicity and efficiency.

**Table 4:** Comparison of Cas12a-based assay with RT-qPCR and NGS

	**NGS**	**RT-qPCR**	**CRIPSR-Cas12a assay**
Plasma isolation	10 min	10 min	10 min
EV isolation	15 min-4 h	15 min-4 h	15 min
DNA preparation	> 9 h	~ 30 min	-
Detection	> 6 h	~ 1 h	~ 5 min
Data analysis	> 8 h	> 10 min	instantaneous
Turnaround time	> 2 day	> 2 h	~ 30 min
LOD of mutation	0.1%-1%	0.1%-5%	~ 1%
Bulky instrument	Yes	Yes	Yes or No
Cost	High	Medium	Low

RT-qPCR: Real-time quantitative PCR; NGS: next-generation sequencing; EV: extracellular vesicle; LOD: limit of detection.

The future scope of EV-based disease detection^[12]^ assays is vast and holds significant promise for advancing cancer diagnostics and personalized medicine. One major direction is broadening the mutation profiles targeted by these assays to include genetic alterations specific to a variety of cancer types, such as *KRAS* mutations for pancreatic and colorectal cancers or *PIK3CA* mutations for breast cancer. By expanding the repertoire of detectable mutations, these assays can evolve into universal diagnostic platforms applicable to multiple malignancies. A combination of Cas12a-crRNAs targeting multiple mutations could enable simultaneous and high-throughput detection of various actionable mutations for EGFR-targeted therapy. The design of this assay holds promise for developing cost-effective, point-of-care devices for bedside diagnostics in clinical environments or even for at-home patient self-testing, facilitating the monitoring of treatment efficacy beyond oncology centers. Another critical area for future development is enhancing the sensitivity and specificity of the assays to detect rare or low-abundance mutations, such as exon 20 insertions in EGFR, which are often associated with treatment resistance. This would ensure their utility in identifying diverse tumor subtypes and guiding targeted therapy decisions. Additionally, the development of high-throughput detection capabilities will enable simultaneous testing for multiple actionable mutations, accelerating the diagnostic process for complex cancer profiles. The integration of EV-based assays into point-of-care devices represents a transformative opportunity for healthcare. Portable and cost-effective diagnostic tools could facilitate bedside or at-home cancer monitoring, greatly improving accessibility for patients and reducing the burden on healthcare facilities. Furthermore, the combination of EV-based detection with other liquid biopsy components, such as circulating tumor cells (CTCs) or circulating free DNA (cfDNA), has the potential to provide a comprehensive picture of the tumor’s genetic landscape^[31,32]^, further enhancing diagnostic accuracy and treatment planning. These assays also have immense potential for early cancer detection by focusing on mutations associated with initial tumor development, thereby increasing the chances of successful intervention. Lastly, their use in longitudinal monitoring of patients offers a way to track treatment responses over time, distinguishing between responders and non-responders and enabling adaptive therapeutic strategies. These advancements could ultimately establish EV-based assays as indispensable tools in the fight against cancer.

In summary, our assay offers a competitive alternative to RT-qPCR and NGS for mutation detection, boosting self-diagnosis and treatment monitoring.

## DECLARATIONS

### Availability of data and materials

All collected, observed, or generated information that validates original research findings is available from the corresponding author upon reasonable request.

### Copyright

© The Author(s) 2025.
